# Genome wide expression profiling of two accession of *G. herbaceum *L. in response to drought

**DOI:** 10.1186/1471-2164-13-94

**Published:** 2012-03-16

**Authors:** Alok Ranjan, Deepti Nigam, Mehar H Asif, Ruchi Singh, Sanjay Ranjan, Shrikant Mantri, Neha Pandey, Ila Trivedi, Krishan Mohan Rai, Satya N Jena, Bhupendra Koul, Rakesh Tuli, Uday V Pathre, Samir V Sawant

**Affiliations:** 1 Council of Scientific and Industrial Research-National Botanical Research Institute, Rana Pratap Marg, Lucknow 226001, UP, India; 2 National Agri-Food Biotechnology Institute, Department of Biotechnology, C-127, Industrial Area, S.A.S. Nagar, Phase 8, Mohali-160071, Punjab, India

## Abstract

**Background:**

Genome-wide gene expression profiling and detailed physiological investigation were used for understanding the molecular mechanism and physiological response of *Gossypium herbaceum*, which governs the adaptability of plants in drought conditions. Recently, microarray-based gene expression analysis is commonly used to decipher genes and genetic networks controlling the traits of interest. However, the results of such an analysis are often plagued due to a limited number of genes (probe sets) on microarrays. On the other hand, pyrosequencing of a transcriptome has the potential to detect rare as well as a large number of transcripts in the samples quantitatively. We used Affymetrix microarray as well as Roche's GS-FLX transcriptome sequencing for a comparative analysis of cotton transcriptome in leaf tissues under drought conditions.

**Results:**

Fourteen accessions of *Gossypium herbaceum *were subjected to mannitol stress for preliminary screening; two accessions, namely Vagad and RAHS-14, were selected as being the most tolerant and most sensitive to osmotic stress, respectively. Affymetrix cotton arrays containing 24,045 probe sets and Roche's GS-FLX transcriptome sequencing of leaf tissue were used to analyze the gene expression profiling of Vagad and RAHS-14 under drought conditions. The analysis of physiological measurements and gene expression profiling showed that Vagad has the inherent ability to sense drought at a much earlier stage and to respond to it in a much more efficient manner than does RAHS-14. Gene Ontology (GO) studies showed that the phenyl propanoid pathway, pigment biosynthesis, polyketide biosynthesis, and other secondary metabolite pathways were enriched in Vagad under control and drought conditions as compared with RAHS-14. Similarly, GO analysis of transcriptome sequencing showed that the GO terms *responses to various abiotic stresses *were significantly higher in Vagad. Among the classes of transcription factors (TFs) uniquely expressed in both accessions, RAHS-14 showed the expression of ERF and WRKY families. The unique expression of ERFs in response to drought conditions reveals that RAHS-14 responds to drought by inducing senescence. This was further supported by transcriptome analysis which revealed that RAHS-14 responds to drought by inducing many transcripts related to senescence and cell death.

**Conclusion:**

The comparative genome-wide gene expression profiling study of two accessions of *G.herbaceum *under drought stress deciphers the differential patterns of gene expression, including TFs and physiologically relevant processes. Our results indicate that drought tolerance observed in Vagad is not because of a single molecular reason but is rather due to several unique mechanisms which Vagad has developed as an adaptation strategy.

## Background

Drought is a major abiotic stress that affects plant growth and reduces plant yield. Many plants have evolved specific adaptive mechanisms in response to the drought stress exhibiting either drought escape or drought-tolerant mechanisms. Drought tolerance is a polygenic trait that involves a cascade of responses ranging from physiological changes to transcriptional regulation. The adaptive mechanisms in response to drought in plants are reduced water loss, reduced radiation absorption, reduced evaporation surfaces, and low tissue water potential. Other adaptive mechanisms include maintaining cell turgor pressure and reduced water loss by the accumulation of compatible solute molecules such as betaine, proline, sorbitol, and so on [1]. The known molecular adaptive mechanisms in response to drought in plants involve the activation of transcriptional regulators such as DREB/CBF, MYB, and MYC [2,3]. The development of microarray-based expression profiling methods have triggered significant progress in the characterization of the plant response to various abiotic stresses. Some recent efforts include identification of the gene networks involved in response to high-temperature stress in developing barley caryopses [4]. Further, the cross-hybridization studies using Rice Gene chip lead to the identification of drought-inducible genes in banana [5]. The dynamics of gene networks that are functional at the reproductive stage in response to drought stress have recently been studied in contrasting barley genotypes [6]. Another technology for transcriptome sequencing that uses Roche's GS-FLX pyrosequencer leads to the identification of several unique genes in rice seedlings exposed to drought stress [7]. Further, pyrosequencing helps in the identification of small RNA [7], microsatellite markers [8], and important agronomic traits [9-11] involved in the adaptation of plants to various abiotic stress conditions. Taken together, microarray and pyrosequencing have enormously contributed to the advancement of knowledge related to various genetic networks involved in adaptation.

Cotton (*Gossypium sp*.) is a leading textile fiber as well as the second most important oil seed crop in the world [12], and its productivity is vulnerable to drought. Molecular studies on cotton in response to drought are scanty. In drought-prone areas in Asia, diploid species *Gossypium herbaceum *(A1-genome) and *Gossypium arboreum *(A2-genome) are cultivated preferentially due to their inherent ability to withstand drought [13]. In the NCBI database, till date, only 662 partial nucleotide sequences and 268 ESTs have been deposited for *G. herbaceum*, which is considerably low considering the genome size of G. *herbaceum*, which is 1.7 Gbp [14]. The present study was undertaken to explore the *G. herbaceum *species with the aim of understanding the different genomic and physiological responses that might be involved in the inherent ability of this species to adapt to drought stress.

## Methods

### Screening of *G. herbaceum *accessions for drought tolerance and sensitivity

Fourteen accessions (Vagad, Gujcot21, RAHS-14, RAHS-IPS 187, H-17, AH-7GP, AH-127, RAHS 127, AH41, DB3-12, RAHS131, Jayelehar, GH18-2LC, and RAHS 132) of *G. herbaceum *that had been collected from different geographical locations in India were analyzed for drought tolerance and sensitivity based on mannitol-imposed drought stress in the DT1 experiment. Fifty seeds were maintained for germination, each at 2%, 4%, 6%, and 8% of mannitol in MS media of fourteen accessions. Accessions that grew and survived in 8% mannitol were considered drought tolerant, and other accessions that grew in only 4% mannitol were considered drought sensitive. In the DT2 experiment, two-week-old seedlings were exposed to drought by withholding water for seven days (soil moisture below 30%), whereas the control pots were irrigated daily. In the DT3 experiment, drought stress was given to the plants by withholding watering till soil moisture reaches below 30% in pots and drooping effects on plant leaves became prominent.

### Field experiment

Vagad and RAHS-14 seedlings were grown during the summer of 2010 under field conditions at the Mahatma Gandhi Mission, Aurangabad, at Padegaon, India (19° 15' N; 75° 23' E, 513 m above sea level), in the rainout experimental station. Ten replicates of both the accessions were grown in three plots each with a surface area of 36 m^2^, filled with 1 m deep black cotton soil. A mobile greenhouse-grade polyethylene roof covered the plants during rainfall, and water was supplied through a trickle irrigation system in each plot. Plants were grown using otherwise normal agricultural practices, that is, plant spacing, fertilizers, and so on. Control plots were irrigated to field capacity twice weekly. Drought treatment was imposed by withholding irrigation. The measurements of different parameters were made after ten days (moderate stress) and after thirty days (severe stress) while withholding water. All gas exchange measurements were made between 8 h and 12 h, as gas exchange and environmental parameters did not show significant diurnal variation during this period.

### Measurement of physiological parameters

Leaf gas exchange net photosynthetic rate (A), stomatal conductance (g_s_), and transpiration rate (E) were measured with an LI-6400 portable photosynthesis system (Li-Cor, Lincoln, NE) with red and blue LED light sources. All measurements were made on the third and fourth leaves from the terminal bud of a twig. Measurements were made between 8 h and 10 h on five leaves per treatment per accession on different plants. Measurements were conducted at c. 400 μmol CO_2 _mol^_1 ^air, constant leaf temperature (T leaf = 33 ± 2°C), and constant vapor pressure deficit (VPD = 2.5 kPa ± 0.2) after the attainment of steady-state photosynthetic rates. The ratio of (A) to (E) was taken as the intrinsic photosynthetic water use efficiency (WUE). Dark respiration (R) was measured under similar microclimatic conditions after dark adaptation of the leaf for more than 30 min. Measurements of water potential and relative water content (RWC) were made at predawn on single, fully expanded leaves (third and fourth leaves from the terminal bud of a twig) immediately after excision. Leaf water potential (Ψ) was measured with a plant water status console (Soilmoisture, Santa Barbara, CA), whereas RWC of the leaf was calculated as 100 × (fresh weight - dry weight)/(turgid weight - dry weight) [15].

### Sample collection and RNA isolation

Two accessions (Vagad and RAHS-14) of *G.herbaceum *were used for this study. Drought stress was given to potted plants by withholding water to maintain the soil moisture always less than 30%. The drought treated plants were watered only once in every alternate week while the control pots were irrigated daily. A total 12 plants were grown in earthen pots, including six plants from each accession. Drought stress, was given to plants by withhold watering in six pots including three from both accession. The drought treatment was given till the visible differences became apparent. Remaining six pots including three pots from both accessions were watered normally and considered as control. Thus, three plants from each accession at given condition were considered as biological replicates. Total RNA were extracted from the leaf tissues using Spectrum plant total RNA Kit, (Sigma-Aldrich) according to the manufacturer's instructions. After DNaseI treatment (Ambion), RNA were quantified and checked for the integrity by using a Bioanalyzer 2100 (Agilent, Inc., Palo Alto, CA, USA).

### RNA labeling and hybridization

The direct labeling procedure was used with 1 μg of total RNA sample; double-stranded cDNA was synthesized with a T7 promoter-containing oligo (dT) primer using a Gene chip one-cycle cDNA synthesis kit (Affymetrix), followed by *in vitro *transcription using a Gene chip IVT labeling kit (Affymetrix). The biotinylated cRNA was fragmented for hybridization to Affymetrix cotton genome arrays and incubated at 45°C temperature for 16 h at 60 RPM in a hybridization oven. Arrays were washed and stained on an Affymetrix Fluidics Station 450. The arrays were scanned using Gene chip Scanner 3000. A summary of the image signal data for every gene interrogated on the array was generated using the Affymetrix MAS 5.0 (GCOS v1.3) statistical algorithm.

### Microarray data analysis

We used Affymetrix Cotton Gene chip and Array Assist Software 5.2.2 (Agilent Technologies, Santa Clara, CA, USA) for comparative gene expression analysis. Raw cel files were exported from GCOS^® ^software using data transfer tools for data processing and analysis in MeV and Array Assist Software 5.2.2 (Agilent Technologies, Santa Clara, CA, USA). Gene expression data analyses were completed using a filtered RMA expression value [16]. Missing values were filtered, normalized, and natural log2 transformed for biological replicates. The t-test was used to determine the statistical significance of the differentially expressed gene. Probe IDs with detection p value ≤ 0.05 in three biological replicates were considered as present. Expression of genes in watered condition was compared between Vagad and RAHS-14 at p value ≤ 0.05 and fold Change (FC) ≥ 2.0. Similarly under drought stress condition expressed genes were analyzed between Vagad and RAHS-14. We have compared microarray data of Vagad and RAHS-14 in control and drought condition. Thus, when we indicate the genes as uniquely expressed in Vagad that means they were up-regulated in Vagad as compared to RAHS-14 and thus those genes were down regulated in RAHS-14 and vice a versa. The cRNA hybridization data were submitted according to MIAME guidelines, which were accessible through GEO series accession number GSE26522 http://www.ncbi.nlm.nih.gov/geo/query/acc.cgi?acc=GSE26522. The statistical analyses were conducted by MeV and Array Assist [17].

### Annotation analyses of cotton Gene chip

Differentially up-regulated genes were analyzed using the functional categorization based on three GO categories at p-values ≥ 0.05. The agriGO tool http://bioinfo.cau.edu.cn/agriGO/ was used to perform the enrichment analysis using SEA (Singular Enrichment Analysis) coupled with available background data of cotton probes. Gene percentage analysis was calculated for each agriGO annotation in the GO category. Cotton Gene chip annotation was based on the top hits against the *Arabidopsis *genome (from TAIR release 8) using the PLEXdb tool and the *Arabidopsis *Genome Initiative databases.

### Double-strand cDNA library preparation for GS-FLX pyrosequencing

Total RNA (3 μg) from apical leaf tissue from both the accessions were reverse transcribed using a T7-Oligo (dT) Promoter Primer in the first-strand cDNA synthesis (Affymetrix). After RNase H-mediated second-strand cDNA synthesis, the double-stranded cDNA was enriched and served as a template in the subsequent *in vitro *transcription (IVT) reaction (Affymetrix). The IVT reaction was carried out in the presence of T7 RNA Polymerase (Affymetrix). The cRNA (3 μg) was reverse transcribed in the first-strand cDNA synthesis step by using a random hexamer primer, followed by RNase H-mediated second-strand cDNA synthesis in replicates. The replicate samples were pooled and purified by the QIAquick PCR purification column (Qiagen) and the purified samples were used for sequencing.

### Emulsion-based clonal amplification and pyrosequencing

Double-strand cDNA was nebulized in a fragment size between 400 and 600 bp. The fragmented cDNA were amplified in aqueous droplets that were made through the creation of a PCR reaction mixture in emulsion oil. The droplets act as separate microreactors in which parallel DNA amplifications are performed while yielding approximately 10^7 ^copies of a template per bead. One microliter of emulsion containing approximately 1.8 thousand beads was prepared. After PCR, the emulsion was broken to release the beads containing the amplified DNA template. The beads carrying the templates were enriched and deposited by centrifugation into open wells of a 70 × 70 mm^2 ^optical picotiter plate. The beads containing a mixture of ATP sulfurylase and luciferase were loaded on the plates to generate light from free pyrophosphate to create the individual sequencing reactors in wells. The light generated from free pyrophosphate was transmitted through the base of the optical picotiter plate and detected by a large-format CCD. The images were processed to yield the sequence information.

### Assembly and annotation of transcriptomes

All sequence analyses was conducted using publicly available software, such as R package http://www.R.project.org, MeV, and custom perl scripts. The quality-filtered reads were assembled at criteria of overlap size 100 bp and percent identity 96% using the CAP3 program. To remove the redundancy within both libraries, blastN was used in both libraries against itself, and the pooled sequences had ≥ 90% identity over the length of 75%. Only the largest sequence in each of these pools was considered. Using these criteria, the sequences obtained were called *exemplars*. The exemplar sequences for both libraries were tagged with the library name and pooled for annotation. For annotation, the pooled exemplars were queried against the NCBI nucleotide database (NT) using blastN at evalue of 10^-10 ^and an alignment length of more than 50% of the query sequence. All the *Gossypium *ESTs available at the NCBI database were downloaded and pooled. The pooled exemplars were also queried against all public Cotton EST databases to identify new transcripts of *Gossypium*. Roche's GS-FLX sequence reads discussed in this article can be found in the Genebank http://www.ncbi.nlm.nih.gov/genbank of the National Center for Biotechnology with accession number SRA029162.

### ESTScan model

To assign the orientation of the transcripts, all the pooled exemplar sequences were analyzed by the ESTScan Model, which is trained on *Arabidopsis *and *Oryza *models. The sequences that passed the ESTScan model were translated according to the frame decided by the ESTScan program. These protein sequences were annotated using the blastP program against the NR Uniprot and pfam databases, at evalue of 10^-10^, and an alignment length of at least 50% of the query length. Gene names were assigned to each sequence based on the best blast hit.

### GO analyses

The GO annotations for the sequences were derived using their Uniprot annotation. The Uniprot database was used, as it had extensive GO mapping. The GO annotation for level 5 was extracted for each library and used for further analysis.

### Digital expression analyses

For the digital expression analysis, the reads for both libraries were tagged and pooled to form a large dataset of 141,722 reads. These reads were assembled using the CAP3 program at an overlap of 100 bp and 80% identity. These reads were assembled into 17,752 contigs. Further, the contigs were filtered to include only those that have more than five reads. We calculated the R statistics for the filtered genes to identify significant differentially expressing genes [18]. To reduce the false discovery rate, only genes with an R value > 9 were considered. These filtered contigs were annotated using blastN against the NCBI nucleotide (NT) database, blastX against the NCBI non-redundant proteins (NR) and the Uniprot database.

### The Quantitative Gene Expression (QGE) analyses

Recently, matrix-assisted lazer desorption ionization time of flight mass spectrometry (MALDI-TOF MS) was adopted for analyzing gene expression [19]. Each PCR reaction was performed with 1 μl diluted cDNA (0.025 ng/μl), 0.5 μL 10x HotStar Taq PCR buffer, 0.2 μL MgCl2 (25 mM), 0.04 μL dNTP mix (25 mM each), 0.02 μL HotStar Taq Polymerase (50 U/μL, Qiagen), 0.1 μL competitor oligonucleotide (5 × 10^-9 ^μM), 1 μL forward and reverse primer (1 μM each) (primers list, Additional file 1), and 2.14 μL ddH2O. The PCR condition was as follows: 95°C for 15 min for hot start, followed by denaturing at 94°C for 20 sec, annealing at 56°C for 30 sec, extension at 72°C for 1 min for 45 cycles, and finally, incubation at 72°C for 3 min. Excess dNTPs were removed from PCR products with shrimp alkaline phosphatase. A mixture of 0.17 μLhME buffer (SEQUENOM), 0.3 μL shrimp alkaline phosphatase (SEQUENOM), and 1.53 μL ddH2O was added to each PCR reaction. The reaction solutions were incubated at 37°C for 20 min, followed by 85°C for 5 min to inactivate the enzyme. Base extension reaction was performed by using 0.2 μL of selected ddNTPs/dNTP mixture (SEQUENOM), 0.108 μL of selected extension primer, 0.018 μL of ThermoSequenase (32 U/μL, SEQUENOM), and 1.674 μL ddH2O. The reaction mixture was kept at 94°C for 2 min, followed by 94°C for 5 sec, 52°C for 5 sec, and 72°C for 5 sec for 40 cycles. The extended reaction product was purified with spectroCLEAN resin (SEQUENOME) to remove salts in the buffer, and 16 μL resin/water solution was added into each base extension reaction. Approximately 10 nL of purified reaction product was dispensed onto a 384-format SpectroCHIP (SEQUENOM). A modified Bruker Biflex MALDI-TOF mass spectrometer was used for data acquisitions from the SpectroCHIP. Mass spectrometric data were automatically imported into the SpectroTYPER (SEQUENOM) database for automatic analysis such as noise normalization and peak area analysis.

## Results

### Analysis of drought tolerance in *G. herbaceum *L. accessions

**Figure 1:**
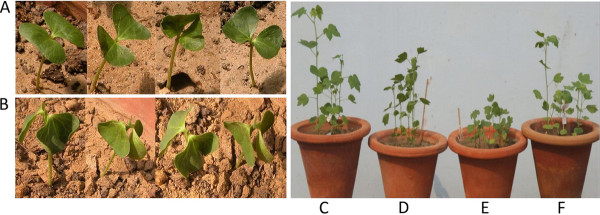
**Effect of drought on cotyledonary stage and one-month-old plants of Vagad and RAHS-14 accessions, (A) Tolerant accession; and (B) Sensitive accession in cotyledonary stage withhold watering for one week; (C) Tolerant accession continuous watering; (D) Tolerant accession; and (E) Sensitive accession of one-week alternate watering; (F) Sensitive accession continuous watering of one-month-old plants**.

*G. herbaceum *accessions were studied for drought tolerance and sensitivity in three experiments: DT1, DT2, and DT3 (details in M&M). In the DT1 experiment, *G. herbaceum *accessions were subjected to mannitol stress in the screen for tolerance to osmotic stress. Among the different accessions, Vagad showed 100% germination of seeds in 6% of mannitol and 86% germination in 8% of mannitol, but RAHS-14 showed only 12% germination in 4% of mannitol and in the case of 6% and 8% of mannitol, the seeds were not germinated at all (Table 1). Accession Gujcot-21 showed 82% and 66% germination of seeds in 6% and 8% of mannitol concentrations, respectively. RAHS-IPS 187 showed only 16% germination in 4% of mannitol, and seeds were not germinated in 6% and 8% of mannitol. Germination of seeds of the remaining accessions was not affected by 4% of mannitol, but a difference was observed at 6% and 8% of mannitol concentrations. In DT2 experiments, the cotyledonary leaves of Vagad seedlings remained green and turgid after seven days of water stress (Figure 1a), whereas RAHS-14 seedlings turned pale and exhibited a drooping effect (Figure 1b). In DT3 experiments, both Vagad and RAHS-14 showed prominent effect of drought stress. However, Vagad showed much better development, less wilting and higher biomass as compared to RAHS-14 (Figure 1c, d), where as RAHS-14 showed stunted growth of plants, more leaf wilting and pale leaves in response to drought stress (Figure 1e, f). In view of the contrasting response of Vagad and RAHS-14, the two accessions were subjected to further physiological and molecular investigation.

**Table 1:** Screening of G. herbaceum accessions at different concentrations of mannitol.

		Mannitol percentage			
**Accessions**	**Control**	**2%**	**4%**	**6%**	**8%**

Vagad	100	100	100	100	86
Guj cot-21	100	100	100	82	66
RAHS-14	100	76	12	0	0
RAHS-IPS-187	100	100	16	0	0
H-17	100	100	84	62	14
AH-7GP	100	100	100	14	0
AH-127	100	100	100	22	4
AH-41	100	100	100	18	0
RAS-45	100	100	100	18	0
DB-3-12	100	100	100	64	30
RAHS-131	100	100	100	16	0
JYLEHAR	100	100	100	14	2
GH-18-2LC	100	100	100	86	22
RAHS-132	100	100	34	16	10

Fifty seeds were kept for germination at 2%, 4%, 6%, and 8% of mannitol concentration in Hoagland media for fourteen different accessions of *G. herbaceum*. The percentage of seed germination was calculated after 2 weeks of growing seedlings

### Analyses of various physiological parameters in response to drought

**Figure 2:**
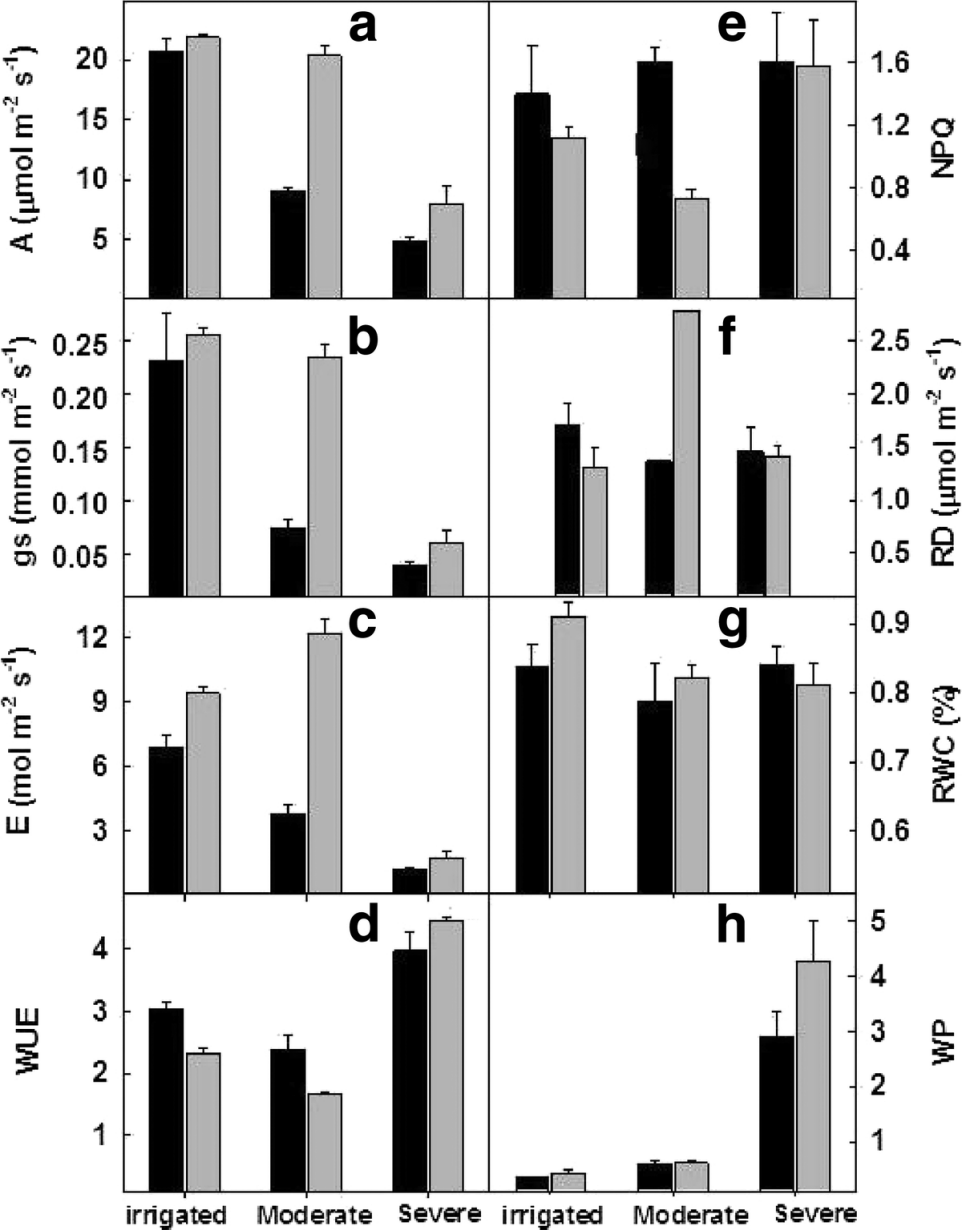
**Effect of moderate and severe drought on (A) Photosynthesis (A, μmol m^-2 ^s^-1^); (B) stomatal conductance (g_s_, mmol m^-2 ^s^-1^); (C) transpiration rate (E, mmol m^-2 ^s^-1^); (D) water use efficiency (WUE mmol CO_2 _mol H_2_O m^-2 ^s^-1^); (E) non-photochemical quenching; (F) dark respiration (RD μmol m^-2 ^s^-1^); (G) relative water content (RWC %); and (H) predawn water potential (ψ_pre _MPa) in Vagad (Black bars) and RAHS-14 (gray bars)**. Data are given as mean values ± n = 5.

**Figure 3:**
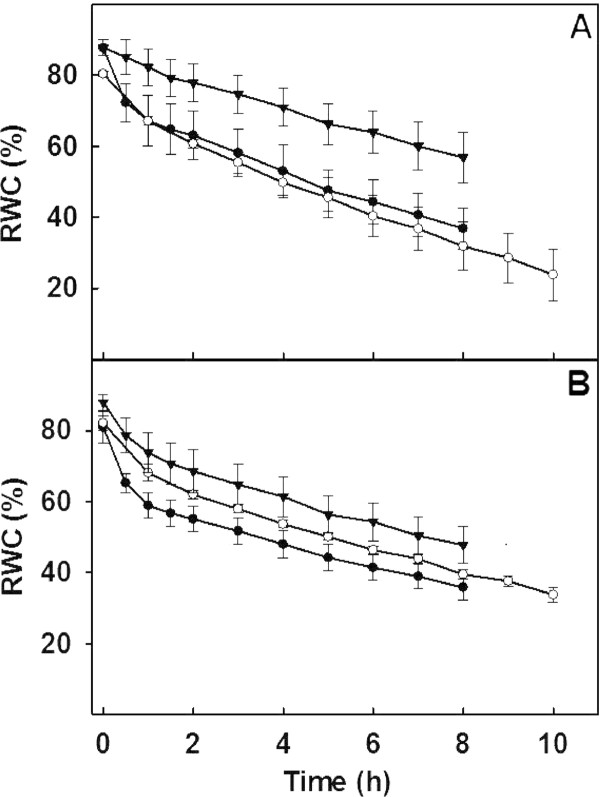
**Effect of moderate (○) and severe drought (●) on leaf dehydration curve in comparison to irrigated plants (▼) of (A) Vagad and (B) RAHS-14 accessions**.

Measurement of gas exchange parameters under irrigated conditions (control) showed marginal differences in the A and g_s _in Vagad and RAHS-14 with RAHS-14 showing slightly higher A and g_s _(Figure 2a, b). However, after 10 days of drought, Vagad showed a sharp decrease in A (> 50%), g_s _(> 75%), and E (> 60%), whereas in RAHS-14, insignificant differences were observed. In RAHS-14, the E was 25% higher than that in Vagad in control plants and further increased after moderate drought (Figure 2c). The WUE was 20% more in Vagad as compared with the RAHS-14 irrigated condition and decreased under moderate drought in both the accessions (Figure 2d). Unlike A, the RD was lower in Vagad compared with that in RAHS-14 and slightly decreased (10%) after moderate drought, but in RAHS-14, the RD was increased almost 2 fold after 10 days of drought (Figure 2f). Vagad showed substantially higher thermal dissipation (NPQ) under irrigated and moderate drought conditions as compared with RAHS-14 (Figure 2e). Predawn water potential in both the accessions was similar, but RWC was higher in RAHS-14 leaves as compared with Vagad in irrigated and water-stressed plants (Figure 2g). Both the accessions showed contrasting results for various physiological parameters under moderate drought conditions; however, when the stress was continued further for 30 days, we observed that both accessions have a similar response to severe drought, except for RWC and predawn water potential. Under severe drought conditions, both the accessions showed a substantial increase in water potential: Vagad showed a 5-fold increase, whereas RAHS-14 showed a 10-fold increase in water potential, but RWC was decreased by only 10% (Figure 2g). Leaf dehydration curves for Vagad and RAHS-14 are shown in Figure 3a, b. The initial sharp drop of curves represents the stomatal transpiration that was closing during leaf dehydration. The steady-state decline under the irrigated condition in both the accessions exhibited a similar slope; however, as drought progressed, the slope in Vagad remained the same under moderate drought and then decreased under severe drought, whereas in RAHS-14, the slope remained constant under irrigated and drought conditions.

### Transcriptional profiling during drought and irrigated conditions in Vagad and RAHS-14

We used Affymetrix microarray and cotton chip for comparative expression profiling of leaves of irrigated and water-stressed plants of Vagad and RAHS-14 at a p value ≤ 0.05 and a fold change (FC) ≥ 2.0. We identified 656 and 535 genes as being differentially up-regulated in Vagad and RAHS-14, respectively, during the irrigated condition (Additional files 2 and 3). Similarly, 430 and 411 genes were differentially up-regulated in Vagad and RAHS-14, respectively, under the drought condition (Additional files 4 and 5). These differentially up-regulated genes were further annotated through the SEA (Singular Enrichment Analysis) method and identified enriched gene ontology (GO) terms. We considered only those GO terms that had been enriched at least twice over background cotton data and observed various significant differences in the metabolic pathways in both sensitive and tolerant accession.

### Singular Enrichment Analysis (SEA) for identification of enriched GO terms in Vagad and RAHS-14 during irrigated condition

**Figure 4:**
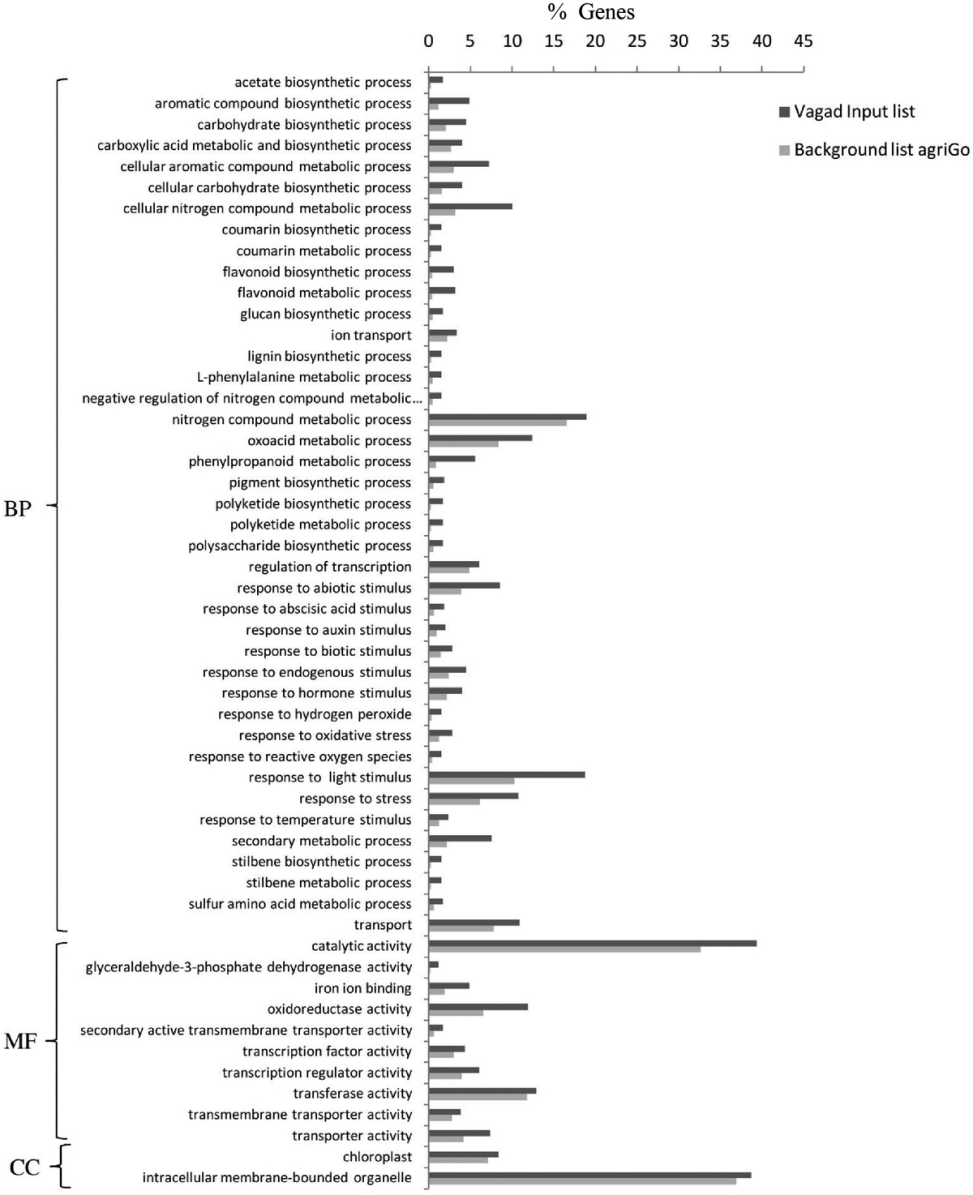
**GO annotation of differentially expressed genes during irrigated condition in Vagad**. BP-Biological process, MF-Molecular function, and CC-Cellular components.

**Figure 5:**
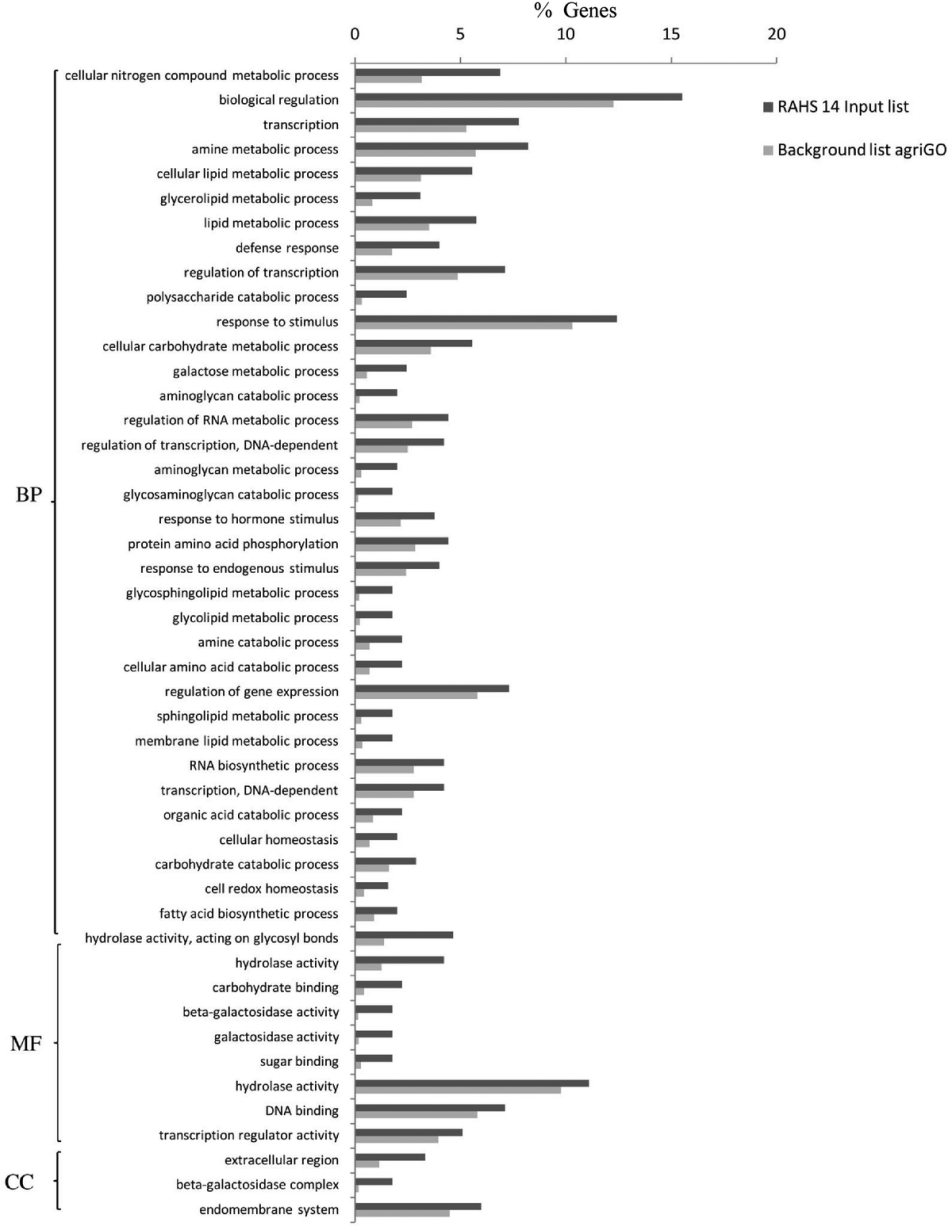
**GO annotation of differentially expressed genes during irrigated condition in RAHS-14**. BP-Biological process, MF-Molecular function, and CC-Cellular components.

Gene ontology using the SEA method revealed distinct differences in the overall metabolism of Vagad and RAHS-14 even under the irrigated condition. Vagad has many enriched biological processes compared with RAHS-14 (Figure 4). The phenyl propanoid pathways leading to coumarin and similarly, a lignin biosynthesis flavonoid pathway leading to polyketide biosynthesis such as stilbene biosynthesis were enriched during the irrigated condition in Vagad (Figure 4). Genes were further mapped to the KEGG metabolic pathway of these processes (Additional files 6 and 7). Other biological processes such as ketone biosynthesis, pigment metabolism, and reductive pentose phosphate were also enriched in Vagad during the irrigated condition. In molecular functions and cellular components, various membrane transporters, ligases, chalcone synthase, glyceraldheyde-3-phosphate dehydrogenase, oxidoreductase, and negative regulation of transcription-related activities were enriched in Vagad (Figure 4). In RAHS-14, in contrast to Vagad, various biological processes such as membrane lipid metabolism involving fatty acid biosynthesis, glycolipid, sphingolipid, and glycosaminoglycan metabolism-related processes were enriched (Figure 5). RAHS-14 also invests its energy in carbohydrate metabolism, various homeostasis-related processes, defense response, auxin metabolism, and root-development-related biological processes (Figure 5). In RAHS-14, molecular function and cellular components were enriched with various activities such as hydrolases, beta-galactosidase, lipases, and esterases.

### Singular Enrichment Analysis (SEA) for identification of enriched GO terms in Vagad and RAHS-14 during drought condition

**Figure 6:**
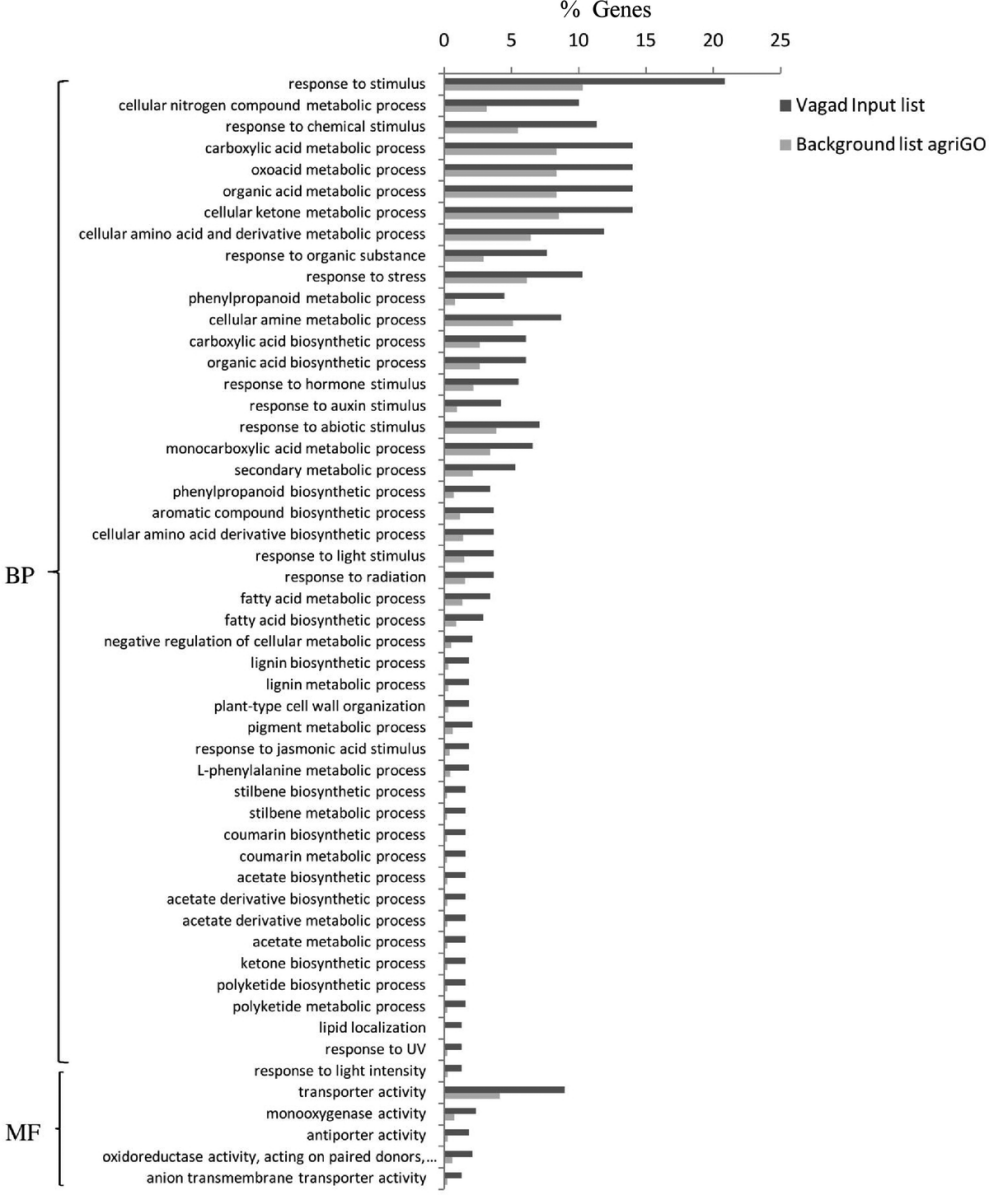
**GO annotation of differentially expressed genes during drought condition in Vagad**. BP-Biological process and MF-Molecular function.

**Figure 7:**
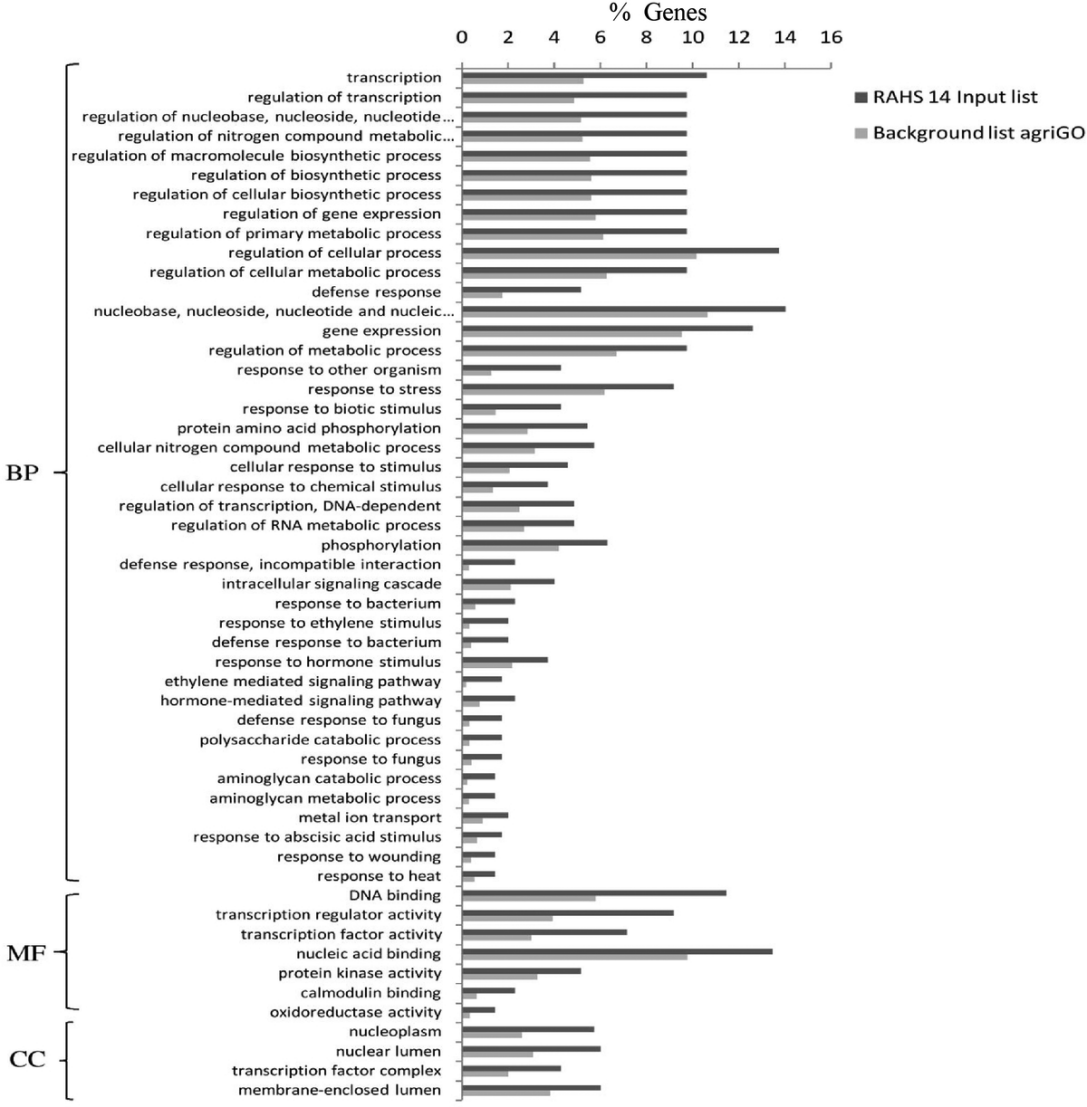
**GO annotation of differentially expressed genes during drought condition in RAHS-14**. BP-Biological process, MF-Molecular function, and CC-Cellular components.

Vagad showed the various biological processes that are involved in the phenyl propanoid pathway, flavonoid pathway, pigment metabolism, polyketide biosynthesis, coumarins, and lignin biosynthesis enriched during drought as observed during the irrigated condition (Figures 4 and 6). In addition, Vagad showed the negative regulation of various forms of cellular biosynthesis and metabolism during the drought condition. Further biological processes involved in response to various stimuli, auxin response, response to light intensity, heat and jasmonic acids, plant cell wall organization, lipid transport, and lipid organization were enriched in Vagad. RAHS-14 showed various biological processes leading to senescence, whereas ABA response and cell death, response to various pathogens, immune response, and response to various hormones were enriched during the drought condition (Figure 7).

### Abiotic-stress-related process analysis of differentially expressed genes

**Figure 8:**
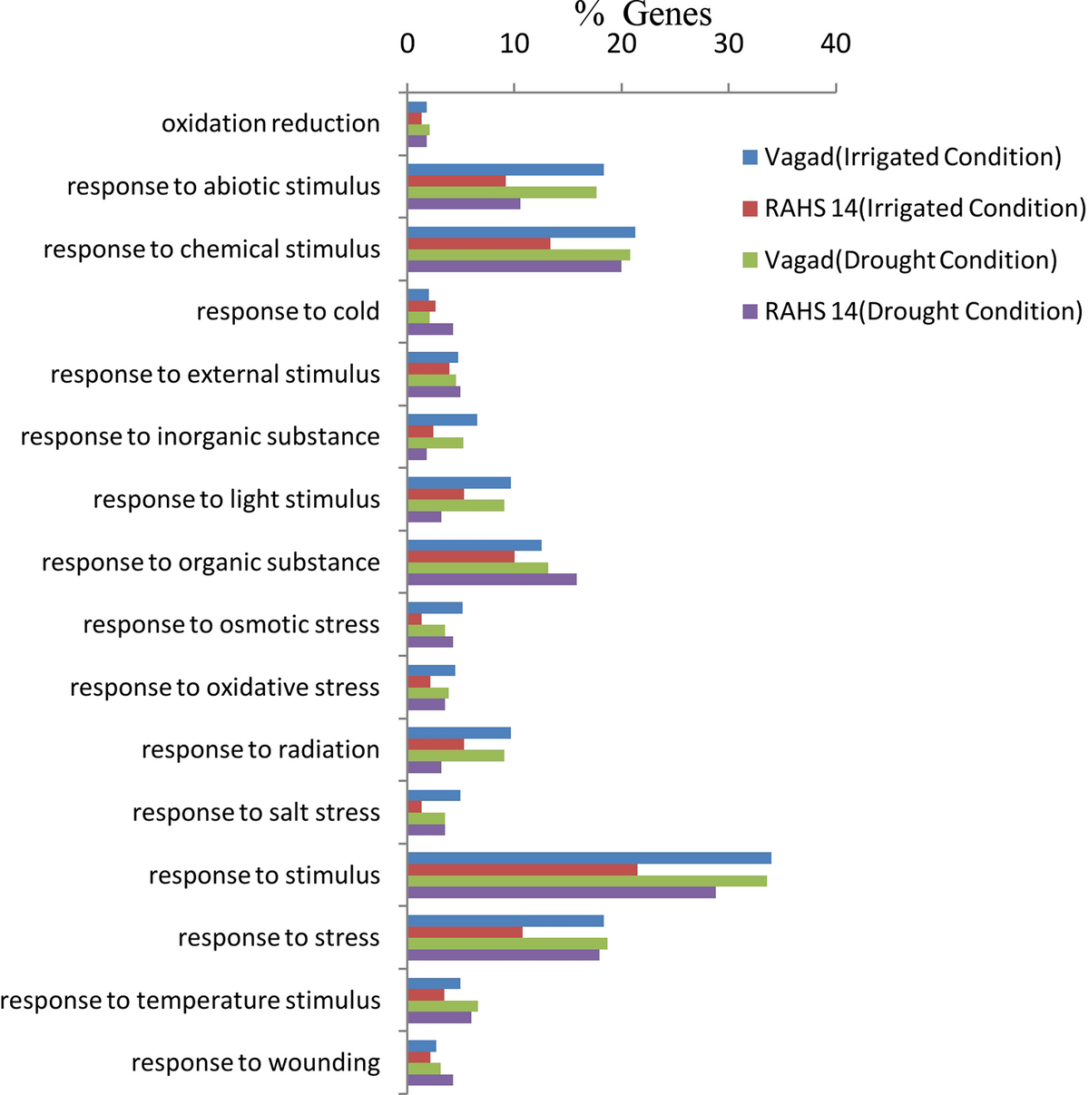
**Comparative analysis of abiotic stress responses in both the accessions under irrigated and drought condition by level 3 GO annotation of differentially expressed genes**.

Gene ontology enrichment analysis http://bioinfo.cau.edu.cn/agriGO/ was performed using an FDR-adjusted p-value of ≤ 0.05 as the cutoff. The distribution of abiotic-enriched GO terms showed several noteworthy findings. In Vagad, almost all the abiotic responses were higher compared with RAHS-14 in irrigated as well as in drought conditions (Figure 8). The significantly enriched GO terms, including *response to abiotic stimulus *(GO: 0009651, FDR *p*-value = 1.40E-03), *response to stress *(GO: 0009737, FDR *p*-value = 3.40E-04), *response to stimulus *(GO:0009723, FDR *p*-value = 1.40E-04), *response to inorganic substances *(GO:0042542, FDR *p*-value = 0.00018), and *response to salt stress *(GO:0006950, FDR p-value = 4.2e-05) showed a significantly higher level in Vagad compared with RAHS-14. The results indicate that the inherent preparedness and responsiveness of Vagad toward drought stress was much higher as compared with RAHS-14.

### RAHS-14 responds to drought by uniquely expressing ERFs (AP2/EREBP) and WRKY

**Figure 9:**
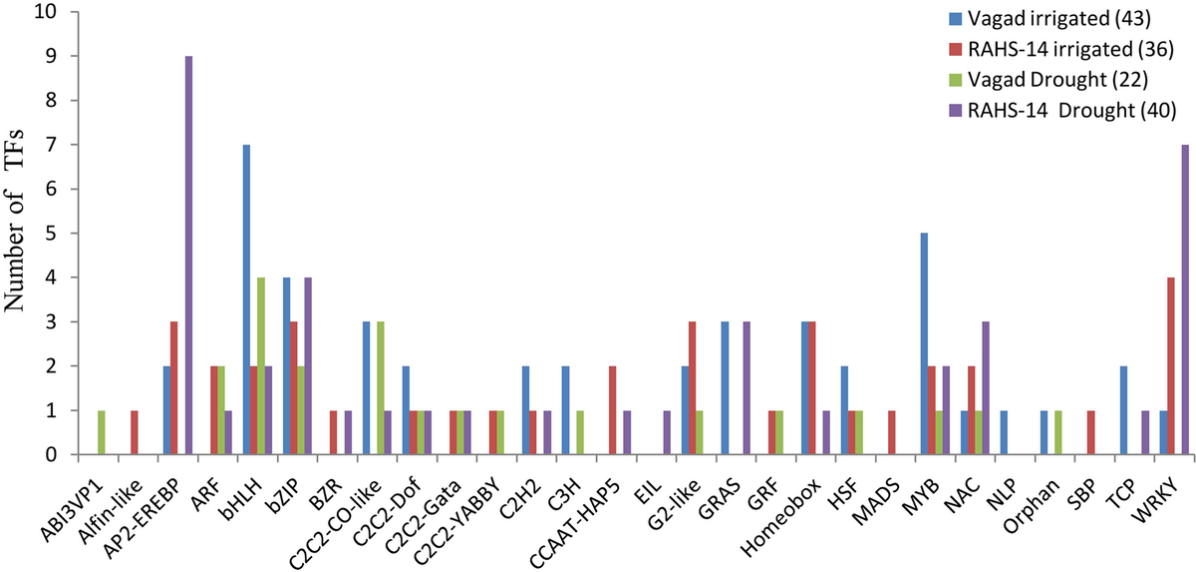
**Differentially expressed TFs under irrigated and drought condition in Vagad and RAHS-14**.

Next, we examined differentially the transcription factors (TFs) expressed in Vagad and RAHS-14 under the irrigated and drought conditions. The homologous locus IDs corresponding to the differentially expressed genes in Vagad and RAHS-14 were queried against the AGRIS database http://arabidopsis.med.ohio-state.edu/AtTFDB/. In the irrigated condition, Vagad and RAHS-14 showed a similar number uniquely expressing TFs, being 43 and 36, respectively (Figure 9); however, in the drought condition, the number uniquely expressing TFs in RAHS-14 was almost double (40) as compared with Vagad (22). The TFs representing all the four categories belong to 28 different classes of TFs, and some of them are uniquely expressed in one or the other categories (Figure 9). Close inspection of the data reveals that AP2/EREBP and WRKY were found to be dominantly expressed in RAHS-14, especially in response to drought. Out of the 9 TFs of the AP2/EREBP family expressed in RAHS-14 in response to drought, 5 belong to the ethylene responsive factors (ERFs) (Additional file 8). In contrast, Vagad showed the expression of only two AP2/EREBP TFs in the irrigated condition belonging to the CRF2 and RAP2.4 class; however, in neither the irrigated nor the drought condition, Vagad showed expression of ERFs. The other most contrasting TFs family found to be dominantly expressing RAHS-14 in the irrigated (4) and drought (7) conditions was WRKY. In Vagad, bHLH and MYB were the two major TFs families found to be dominantly expressing in the irrigated condition. Thus, differences in the expression of the unique TFs families in Vagad and RAHS-14 may reflect the manner in which these two accessions differ in their response to drought.

### Validation of identified significant genes by quantitative gene expression (QGE)

**Figure 10:**
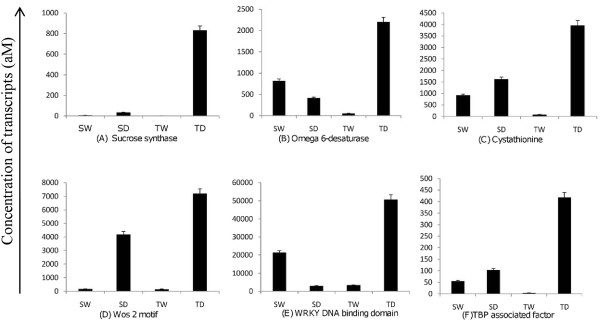
**Gene expression profiling of up-regulated genes in microarray analysis, validated by quantitative gene expression (QGE) during irrigated and drought condition in both the accessions**. (A) Omeg-6-desaturase; (B)Sucrose synthase; (C) Cystathionine; (D) Wos 2 motif; (E) TBP-associated factor; and (F) WRKY DNA binding domains. SD represents RAHS-14 in drought, and SW represents the irrigated condition. TD represents Vagad under drought, and TW represents the irrigated condition.

The six genes identified by microarray analysis as being commonly up-regulated during the drought condition are omega-6-desaturase, sucrose synthase, cystathionin, wos2 motif containing protein, putative TAF-like protein, and a WRKY transcription factor, and they were validated using Quantitative Gene Expression (QGE) assay using SEQUENOM (see M&M). The QGE was performed with three biological replicates for Vagad and RAHS-14 on drought and irrigated samples. The expressions of all the six genes were significantly higher in Vagad during drought as compared with the irrigated samples (Figure 10a-f). Similarly, in RAHS-14, the expression of omega-6-desaturase, cystathionin, [20] wos2 motif containing protein, and putative TAF-like protein was higher during drought as compared with the irrigated condition. However, contrary to the microarray data, the expression of sucrose synthase and WRKY was found to be down-regulated in RAHS-14 in response to the drought condition.

### The transcriptome assembly and annotation

Vagad and RAHS-14 were taken for further analyses by transcriptome sequencing under drought stress by Roche's GS-FLX pyrosequencer. The total numbers of quality-filtered reads obtained were 85638 and 56354 from the leaves of Vagad and RAHS-14, respectively. The reads from both the transcriptome sequences were assembled into contigs and singletons (Table 2) using the CAP3 assembly program (overlap size of 100 bp and 96% identity). Under this stringent criterion, on an average, 65% of the reads were assembled into the contigs. The average lengths of the assembled contigs and singletons were nearly 350 bp and 180 bp, respectively. The number of contigs greater than 500 bp in length was 946 in Vagad and 705 in RAHS-14. The average length of the large contigs was 740 bp. The distribution of reads per contig is presented in Additional file 9. The average depth of the contigs in both the libraries was about five reads per contig. The assembled contigs and singletons were pooled and queried against the NCBI NR database using the blastN program at a stringency of evalue of 10^-10 ^and a greater than 50% overlap of both the query and the subject. At these criteria, 21,179 genes were annotated (Additional file 10). To find the common sequences between already reported cotton ESTs and our unigenes, we queried the dataset against all publicly available cotton ESTs, at criteria of evalue of 10^-10^, and at least 50% alignment of either the query or the subject. This leads to identification of 30,133 sequences matching to the cotton ESTs (Additional file 11); 4946 sequences did not have any match to the cotton ESTs and could be novel sequences specific to *G. herbaceum*. For assessing the full-length transcripts, the pooled contigs and singlets that comprised the unigene dataset were screened using the ESTScan program. Both *Oryza *and *Arabidopsis *gene models were used to train the program (Additional file 12). Out of the total unigene datasets, the numbers of the gene models obtained were 16,283 in Vagad and 14,885 in RAHS-14. Both the libraries were annotated by the blastX program against the Uniprot database, at criteria of 50% alignment length, and evalue of 1 × 10^-10 ^(Additional file 13; worksheet 1 and 2). The ESTScan passed 32.9% genes and was annotated using a Uniprot database. Approximately 20% of the large contigs from both the libraries were unannotated when compared against the Uniprot and NCBI NR database.

**Table 2:** Summary of 454 transcriptome sequencing data generated for Vagad and RAHS-14 of *G. herbaceum *leaves transcriptome and assembly

Parameters	Vagad	RAHS-14
Total reads (overlap size of 100 bp and 96% identity)^a^	85368	56354
Total contigs (100 bp or greater)^b^	11439	6313
Singleton	24087	20780
Exemplar	31244	23155
Average length of contigs	350 bp	180 bp
Number of contigs with greater than 500 bp	946	705
^c^Number of genes with significant hits in NCBI NR database	10772	10408
^d^Number of genes with significant hits in cotton EST database	16301	13822

^a^Total number of reads separated for both transcriptome libraries. ^b^Contigs generated by CAP3 assembly. ^c^Contigs showing significant hits (evalue 10^-10 ^and ≥ 50% overlap) in the NCBI database. ^d^Contigs showing significant hits (e value 10^-10^, and ≥ 50% alignment of either the query or the subject) in the cotton EST database

### GO annotation of transcriptome

**Figure 11:**
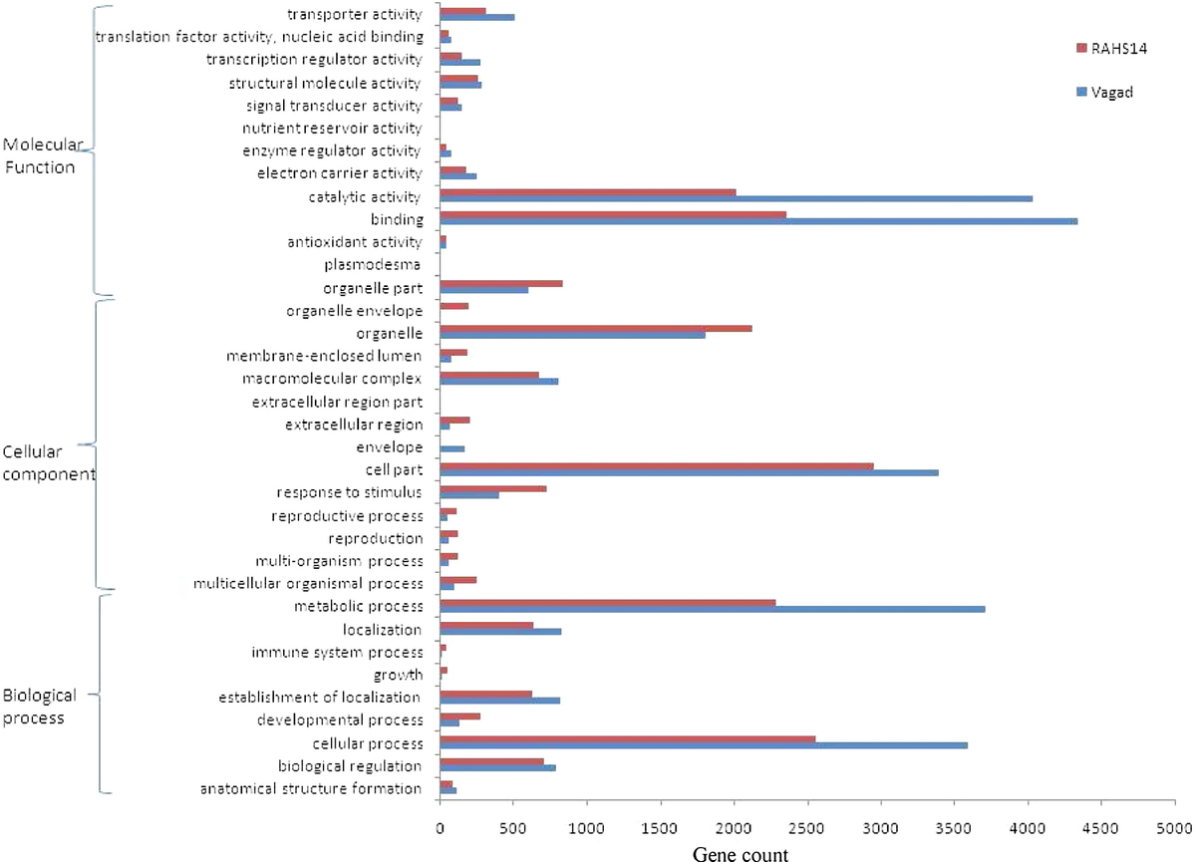
**GO-based annotation of the transcriptome analysis of Vagad and RAHS-14 under drought stress**.

The GO annotation was obtained from the Uniprot accession numbers, and GO classification for five levels was obtained. The GO annotation was plotted for level three from both the libraries (Figure 11). For most of the categories, the gene counts were similar in both the libraries. However, genes related to catalytic activity, binding, cellular and metabolic processes were significantly higher in Vagad, whereas GO categories, such as response to stimulus, reproductive processes, reproduction, multi-organism processes, multi-cellular organism processes, developmental processes, and organelle parts, were significantly higher in RAHS-14.

### Differential gene expression analyses of transcriptomes

For differential expression analysis of the genes in both the transcriptome libraries, the reads from both the libraries were tagged and pooled to form one large dataset that was assembled into contigs using the CAP3 program (overlap length ≥ 100 bp and 80% identity). The 1,41,722 reads clustered into 17,752 contigs and resulted in 13,586 genes for the expression analysis. Significant changes in gene expression were calculated using R statistics (R value ≥ 9) (Additional file 14) and resulted in 2,026 genes, which seemed to be differentially regulated, and their differential expression was analyzed using Pearson uncentered correlation [18]. Differentially expressed genes were annotated using the NCBI NR database (50% alignment length, evalue of 1 × 10^-10^). For each contig, the counts were converted to transcripts per million, which was transformed [log_2 _(fold change values)], and their ratio was calculated for fold changes between drought-tolerant and drought-sensitive tissues. A total of 275 contigs showed a very high expression in Vagad with nearly 49% (137) showing no hits to any proteins in the database (NR, NT, and Uniprot), and 10% genes were either hypothetical or putative expressed proteins, as they showed significant changes in expression. The remaining genes such as ascorbate peroxidase, cysteine protease, delta tonoplastic intrinsic proteins, LEA proteins, and so on that were related to drought stress were up-regulated (Additional file 15 worksheet 1). In RAHS-14, out of 484 genes, only 80 (15%) showed no hits to any protein or nucleotide in the database (NR and NT), and 18% were hypothetical proteins. The remaining 36% annotated genes were from the photosynthesis pathway, with a high expression of Rubisco activase, photosystem II D, and chlorophyll a\b binding proteins. The senescence-associated proteins constituted about 4% of the differentially expressed genes. Other up-regulated genes were cytochrome p450, heat shock protein 90, methionine synthase, and so on (Additional file 15 worksheet 2).

## Discussion

To analyze the differences in the drought tolerance of the *G. herbaceum *accessions at physiological and molecular levels, the plants were exposed to moderate and severe drought stress. It was observed that Vagad and RAHS-14 showed substantial differences in several physiological parameters and relative gene expression in response to drought. Vagad responded to moderate and severe drought by a gradual decline in A, g_s_, and E and, thus, has better WUE (Figure 2). In contrast, RAHS-14 had higher A and g_s _and, thus, lower WUE, which continued under moderate drought and then declined under severe stress (Figure 2a-d). In Vagad, earlier stomatal closure (Figure 2b) provides a large safety margin against embolism formation as observed in several species [20-22]; rather, plants are capable of pre-empting the water stress-induced xylem cavitation by closing their stomata [23-25]. Further, a reduction in A and a higher NPQ in Vagad accession under drought stress suggested that stress had been imposed and hypothesized that antioxidant defense systems and secondary metabolic pathways would have been enhanced in response. Indeed, our microarray expression analysis results showed that in Vagad, various primary and secondary metabolic pathways were enhanced (Figure 5). Flavonoid biosynthesis pathways especially lead to the formation of xanthophyll and finally convert it into anthocyanin, antheraxanthin, and zeaxanthin, which allows the quenching of excess energy from chlorophyll before it reaches reaction centers [26]; the transcript representing flavonoid biosynthesis pathways was found to be enriched in Vagad (Figure 5). In contrast, the xanthophyll-cycle conversion state was lower in RAHS-14 (Figure 2e and Additional file 7). Concurrent to physiological data, transcriptome analysis showed a higher expression of the vitamins metabolic process, such as α-tocopherol in Vagad (Additional file 13; worksheet 1). The α-tocopherol is a powerful antioxidant that scavenges and prevents the formation of free radicals and prevents lipid peroxidation, thereby resulting in damage to thylakoid and chloroplast membranes [27]. Besides flavonoid pathways in Vagad, various other metabolic pathways, including polyketide biosynthesis, phenyl propanoid biosynthesis, and shikimate pathways, synthesize protective molecules such as stilbene, coumarins, and ligninis and are enriched in Vagad, which might help in the detoxification of free radicals and give an advantage to Vagad for surviving in drought stress (Figure 4). These secondary metabolites, particularly phenylpropanoid, were widely reported for their multiple function in response to various forms of environmental stress [28]. The genes related to the shikimate and phenylpropanoid pathways have been reported to express a higher level in drought-tolerant tomato cultivars (*Solanum lycopersicum L*.) as compared with drought-sensitive tomato cultivars [29]. In RAHS-14, the lipid metabolism processes were significantly higher (Figure 5). In water-deficit conditions, the membranes are the main targets of the degradative process, resulting in the formation of polar and non-polar lipid molecules [27]. Thus, the higher lipid metabolic processes in RAHS-14 probably reflect higher membrane degeneration and, hence, the necessity of higher lipid metabolism for membrane homeostasis correlated with their susceptibility of drought stress. Vagad, in contrast to RAHS-14, maintained lipid metabolism and membrane integrity to resist the drought stress. RAHS-14 showed a higher expression of genes related to nucleic acid (DNA/RNA) metabolism, whereas the expression of these genes remains unchanged in Vagad (Additional file 5 and Figure 6). Higher nucleic acid metabolism in RAHS-14 might show an interesting mechanism related to the energy state of the cell that is represented by the salvage pathways [30]. Phosphoribosyl-1-pyrophosphate, a key intermediate component for the synthesis of ribose-5-phosphate in nucleic acid metabolism, showed higher expression in RAHS-14 as representing the operation mechanism of salvage pathways and helps RAHS-14 keep the energy pools from being used up too quickly (Additional file 13; worksheet 2). It indicated that RAHS-14 responds to drought by inducing energy-consuming processes, whereas Vagad has various inherent primary and secondary metabolic processes that maintain growth, albeit slow even under drought. This is further evident from dark respiration (R) data which show that R declined in Vagad and increased in RAHS-14 during drought (Figure 2h). Many genes that were up-regulated in response to drought stress in Vagad are reported to be involved in multiple mechanisms that may contribute to drought tolerance (Additional files 4 and 15, worksheet 1). For example, Aquaporins (AQP), a water-selective channel protein, known to mediate and regulate rapid transmembrane water flow during a wide range of stress response, stomatal movement, and water channel movements, was expressed at a higher level in Vagad (Additional file 4) [31]. Similarly, many tonoplastic intrinsic proteins (TIPs) from maize, *Arabidopsis*, and radish are known to control water exchange between cytosol and vacuole in salt stress and drought stress [32] and these genes were expressed at a higher level in Vagad (Additional file 4). DnaJ heat shock protein/chaperone and Delta 1-pyrroline-5-carboxylate synthase 2 (P5CS2), which enhance root biomass, flowering, and seed setting during abiotic stress [33], were expressed at a higher level in Vagad (Additional files 2 and 3). Yet another significantly induced gene in Vagad was the ERF/AP2-type transcription factor (RAP2.1), which was reported to be induced in drought and cold via an ABA-independent pathway [34]. RAHS-14 expressed more senescence-related genes, whereas we found that in Vagad, genes related to alcohol dehydrogenase and late embryogenesis protein 5 was expressed at a higher level (Additional files 5 and 15). Previous studies indicate that the accumulation of late embryogenesis abundant proteins and alcohol dehydrogenase (Adh) gene are correlated with stress tolerance [35,36]. The significant up-regulation of LEA and Adh genes in Vagad in drought stress suggests that these genes play an important role in conferring drought tolerance, whereas RAHS-14 tends toward the senescence during drought stress. Several transcription families were significantly and differentially expressed in both the accessions. Ethylene-responsive element binding factors (ERFs) are members of a novel family of transcription factors that are specific to plants and which regulate nuclear gene expression under various stress conditions. Six different members homologous to the *Arabidopsis *ERF family (At1g19210; At1g28360; At4g34410; At5g44210; At5g47220; At5g47230) were expressed exclusively in RAHS-14 under drought stress. The expression of ERF in RAHS-14 in the drought condition indicates that RAHS-14 responds to drought mainly by the ethylene pathway, thereby leading to senescence. This relates well with transcriptome data showing a higher level of senescence-related transcripts in RAHS-14. In Vagad, these AP2/EREBP TFs belong to the Cytokinin Response Factors (CRF2) class (At1g78080; At4g23750). CRFs function redundantly to regulate the various metabolic functions, including transpiration, stomatal conductance, and respiration [37,38]. Further, in RAHS-14, seven (At1g29860; At1g80840; At2g23320; At2g24570; At2g38470; At3g56400; At4g24240) and four (At1g80840; At2g47260; At4g24240; At5g49520) WRKY transcription factors were found to be uniquely expressed in drought and irrigated conditions, respectively. Previous reports showed that abscisic acid and salicylic acid have been involved in the WRKY-mediated hormone signal pathway during abiotic and biotic stress [39]. Their precise role in the abiotic stress response regulatory network is not fully understood [40]. In contrast, the MYC-type bHLH transcription factor, which regulates the expression of CBF3/DREB1A in abiotic stress, was enriched in Vagad, which suggests the involvement of the MYC-type bHLH transcription factors in the expression of CBF/DREB1 genes in Vagad but less involvement in RAHS-14 [41]. Interactions between CBF/DREB1 genes and bHLH TFs and their involvement in various oxidative-mediated processes justify their presumed roles as regulators of drought response in Vagad. Other members of TFs, such as bZIP (At2g46270; At4g34590; At5g24800) and GRAS families (At5g48150; At5g52510; At5g66770), were expressed in higher numbers in Vagad; the regulatory roles of these TFS have been reported in stress responses in plants. The GO-based analysis of both the transcriptome libraries revealed many metabolic processes and responses to various forms of abiotic stress that were specific to Vagad and RAHS-14 (Figure 11). The shift in the processes toward reproductive growth and senescence in RAHS-14 clearly showed that they had crossed the threshold of stress and were proceeding toward senescence. The up-regulation of several metallothioneins, lipid transfer proteins, lea proteins, sucrose synthase, and so on in Vagad showed the induction of defense and stress-related genes to combat drought stress. In addition, the down-regulation of many photosynthesis-related genes during drought stress (Additional file 15 worksheet 2) can be attributed to the conservation of energy for the survival in drought stress. The up-regulation of genes such as aquaporins, lea, and metallothioneins may have played a major role in imparting tolerance to Vagad.

We further compared differentially expressed genes obtained by microarray and contigs obtained in transcriptomic data using Pearson uncentered correlation. A total 167 differentially expressed genes obtained in microarray showed very high Pearson correlation coefficient of 0.845 with transcriptomic contigs obtained under drought stress condition (Additional file 16). Out of these 167 differentially expressed genes, 78 and 48 genes were uniquely represented in Vagad and RAHS-14 respectively under drought stressed condition. Uniquely represented genes under drought stress in Vagad were mainly involved in synthesis of membrane and cytoskeleton associated proteins, oxidoreductases, kinases, heat shock proteins, sugar alcohols and secondary metabolites like, cumurin and stilbene. Similarly in RAHS-14, these genes were mainly associated with transcription factors (viz, WRKY, AP2, WD40, Zinc finger and ERF), senescence associated proteins, ethylene and auxin responsive elements (Additional file 17). The GO based analysis of these 167 genes revealed many processes that were specific to Vagad and RAHS-14. The upregulation of pyrroline-5- carboxylase, ATPase, inositol etc. (Additional file 17) in Vagad may have played a major role in imparting drought tolerance. In RAHS-14 higher numbers of these genes were involved in senescence and ethylene mediated signaling which clearly indicate the RAHS- 14 crossed the threshold of drought stress and are proceeding towards senescence (Additional file 15). Thus both the method in an unbiased way identifies same mechanistic model operating for drought responsiveness in Vagad and RAHS 14.

## Conclusion

We conclude that drought-tolerant accessions such as Vagad must have developed multiple mechanisms as adaptive behavior against drought. These mechanisms are interlinked and probably cannot be seen in isolation; understanding these mechanisms will be helpful for developing our future drought-tolerant varieties.

## Abbreviations

cDNA: Complementary deoxyribonucleic acid; IVT: In vitro-transcription; cRNA: Complementary ribonucleic acid; FC: Fold change; FDR: False detection rate; SEA: Singular enrichment analysis; GO: Gene ontology; EST: Expressed sequence tag; QGE: Quantitative gene expression; CCD: Charge couple device; WUE: Water use efficiency; RD: Dark respiration; NPQ: Nonphotochemical quenching; RWC: Relative water content.

## Competing interests

The authors declare that they have no competing interests.

## Authors' contributions

AR carried out stress treatment and sample collection, design and execution of microarray, transcriptome sequencing, QGE analysis, data integration, and drafted the manuscript. DN analyzed microarray data. MAH and SM analyzed transcriptome data. RS and SR performed physiological experiment, UVP designed and monitor physiological and analyzed the data. NP helped in drafting and revising the manuscript. IT, KMR, SNJ and BK assisted in sample collection, screening and other experimental help. SVS was responsible for the overall concept, designing of problem and experiments, coordination among groups, data analysis and drafting and revising the manuscript. RT mentored the entire project, critical discussion and suggestions. All authors read and approved the manuscript.

## Supplementary Material

Additional file 1 **List of primers for QGE assay**. Excel file containing all primer sequences used for the QGE experiment. Click here for file

Additional file 2 **Annotation and fold change (fold change > = 2) of up-regulated unique genes in Vagad during irrigated condition**. Excel file containing the list of unique up-regulated genes of Vagad during irrigated condition. Click here for file

Additional file 3 **Annotation and fold change (fold change > = 2) of up-regulated unique genes in RAHS-14 during irrigated condition**. Excel file containing the list of unique up-regulated genes of RAHS-14 during irrigated condition. Click here for file

Additional file 4 **Annotation and fold change (fold change > = 2) of up-regulated unique genes in Vagad during drought stress**. Excel file containing the list of unique up-regulated genes of Vagad during drought condition. Click here for file

Additional file 5 **Annotation and fold change (fold change > = 2) of up-regulated unique genes in RAHS-14 during drought stress**. Excel file containing the list of unique up-regulated genes of RAHS-14 during drought condition. Click here for file

Additional file 6 **Phenylpropanoid biosynthesis pathways analysis by KEGG using differentially up-regulated genes in Vagad in drought condition**. JPEG image file containing the pathways mapping of phenylpropanoid biosynthesis from differentially up-regulated genes in Vagad in drought condition. Red color highlighted steps in pathways show involvements of genes in pathways from input gene list. Click here for file

Additional file 7 **Flavonoid biosynthesis pathways analysis by KEGG using differentially up-regulated genes in Vagad in drought condition**. JPEG image file containing the pathway mapping of flavonoid biosynthesis from differentially up-regulated genes in Vagad in drought condition. Red color highlighted steps in pathways show involvements of genes in pathways from input gene list. Click here for file

Additional file 8 **Analysis of differentially expressed TFs**. Excel file containing the summary result of expressed TFs number in both the accessions under drought and irrigated condition. Arabidopsis homolog IDs were mapped from TAIR10. Click here for file

Additional file 9 **Histogram of frequency of the number of reads assembled in contigs**. In JPEG image file, X-axis represents the number of reads, and Y-axis represents the number of genes. The color code indicates the contigs of Vagad and RAHS-14. Click here for file

Additional file 10 **BlastN analysis of both transcriptome contigs and singlets against the NCBI NR database**. Excel file containing summary result of BLAST analysis and short description. Click here for file

Additional file 11 **BlastN analysis of both transcriptome contigs and singlets against publicly available Cotton EST sequences**. Excel file containing summary of BLAST analysis from cotton EST. Click here for file

Additional file 12 **Percentage of contigs passing the ESTScan model in both libraries**. JPEG image showed the total number of EST that has passed through the ESTScan model. Click here for file

Additional file 13 **Uniprot analyses of Vagad and RAHS-14**. Excel file containing uniprot analysis of Vagad in worksheet 1 and RAHS-14 in worksheet 2. Click here for file

Additional file 14 **Differentially expressing contigs that were filtered by an R value of 9**. Excel file containing differentially expressed contigs in Vagad and RAHS-14 and EST counts. Click here for file

Additional file 15 **Fold change gene analysis by digital transcriptome of both the accessions**. Excel file containing short description and fold change of both the accessions. Worksheet 1 showed up-regulated contigs, and worksheet 2 showed down-regulated contigs in Vagad compared with RAHS-14. Click here for file

Additional file 16 **Correlation analysis between differentially expressed genes obtained in microarray and contigs obtained from transcriptome sequencing**. PPT file containing Pearson correlation graph between microarray and contigs of transcriptome sequencing. For each contigs the counts were converted to transcripts per million which was then converted to log2 counts and their ratio was calculated for fold change between Vagad and RAHS-14. Click here for file

Additional file 17 **Annotation analysis of 167 gene obtained in correlation analysis of microarray and transcriptomic contigs**. Excel file contacting detail analysis of differentially expressed genes obtained in microarray correlated with transcriptomic contigs. Click here for file
