# Supplementary figures and images for: Genome wide expression profiling of two accession of G. herbaceum L. in response to drought
Source: BMC Genomics. 2012 Mar 16;13:94. doi: 10.1186/1471-2164-13-94 (PMC3320563; doi:10.1186/1471-2164-13-94)

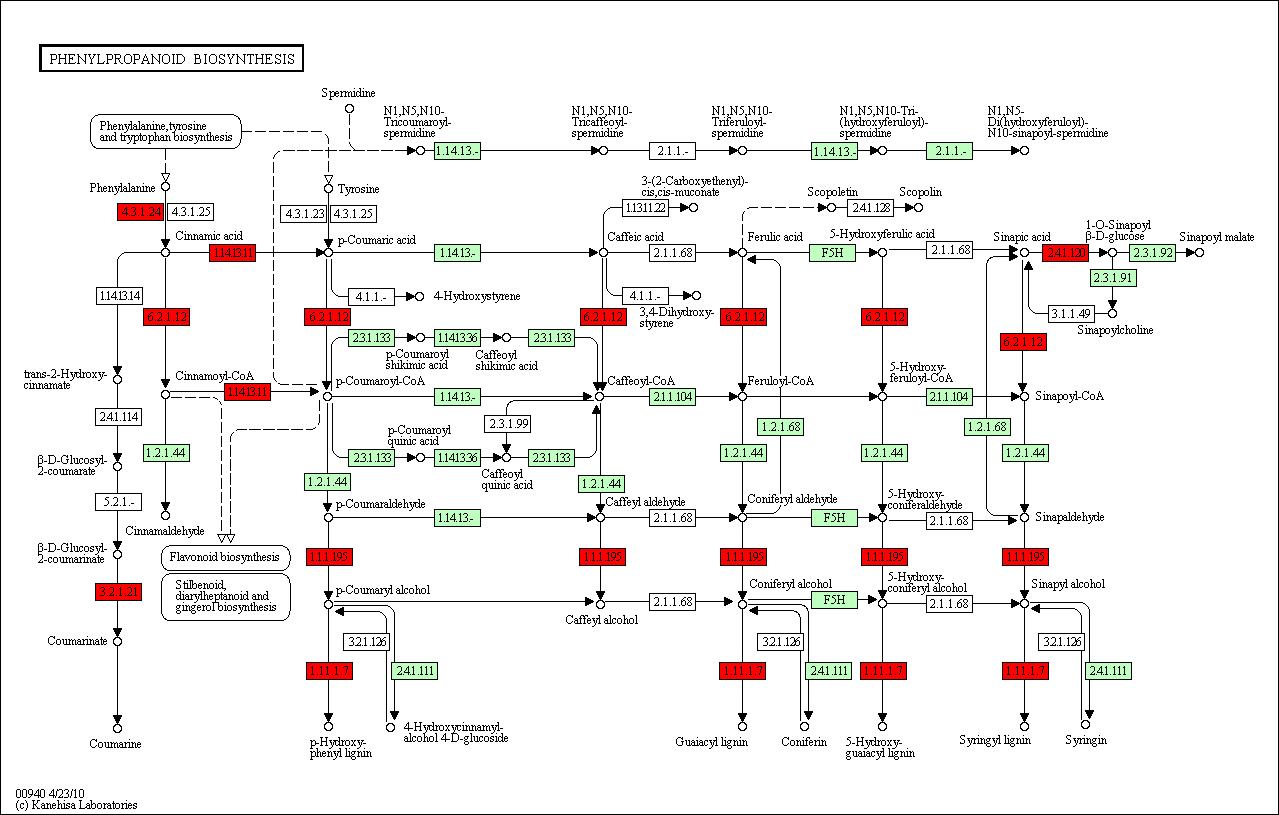

Supplement: Additional file 6 — Phenylpropanoid biosynthesis pathways analysis by KEGG using differentially up-regulated genes in Vagad in drought condition. JPEG image file containing the pathways mapping of phenylpropanoid biosynthesis from differentially up-regulated genes in Vagad in drought condition. Red color highlighted steps in pathways show involvements of genes in pathways from input gene list. [file 1471-2164-13-94-S6.JPEG]

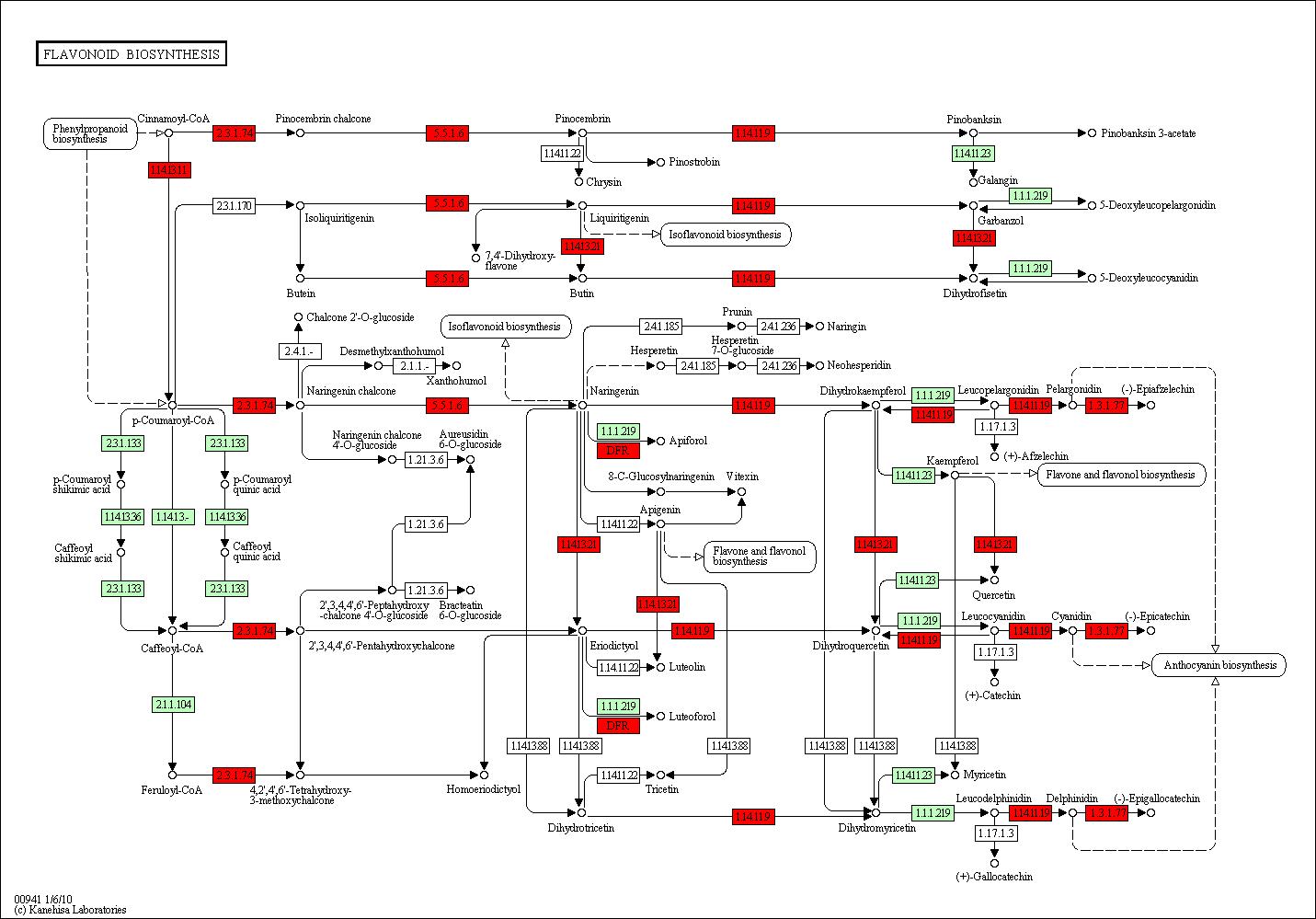

Supplement: Additional file 7 — Flavonoid biosynthesis pathways analysis by KEGG using differentially up-regulated genes in Vagad in drought condition. JPEG image file containing the pathway mapping of flavonoid biosynthesis from differentially up-regulated genes in Vagad in drought condition. Red color highlighted steps in pathways show involvements of genes in pathways from input gene list. [file 1471-2164-13-94-S7.JPEG]

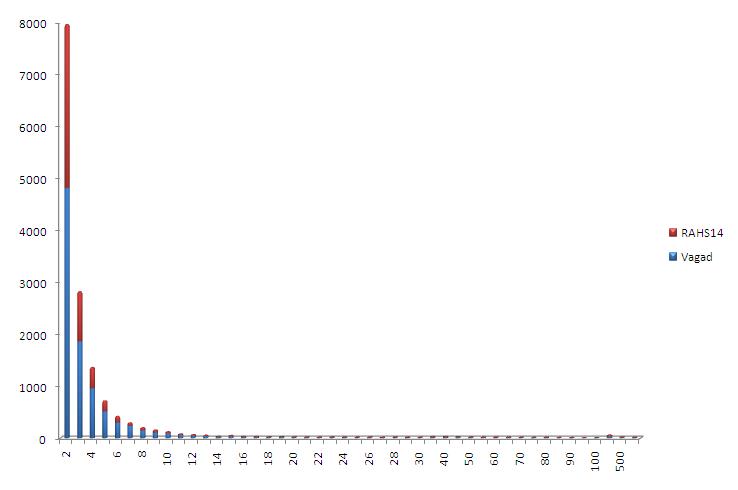

Supplement: Additional file 9 — Histogram of frequency of the number of reads assembled in contigs. In JPEG image file, X-axis represents the number of reads, and Y-axis represents the number of genes. The color code indicates the contigs of Vagad and RAHS-14. [file 1471-2164-13-94-S9.JPEG]

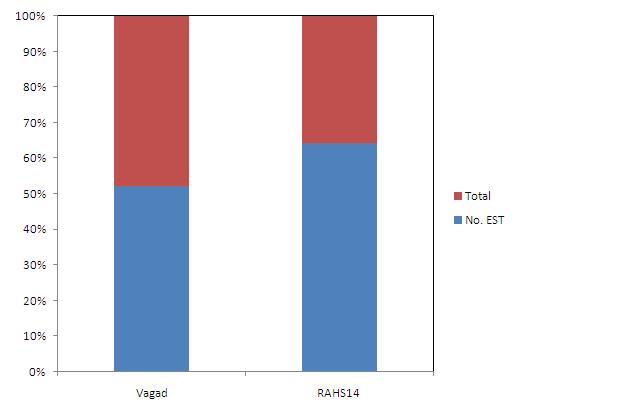

Supplement: Additional file 12 — Percentage of contigs passing the ESTScan model in both libraries. JPEG image showed the total number of EST that has passed through the ESTScan model. [file 1471-2164-13-94-S12.JPEG]
