# Supplementary material for: Genome wide expression profiling of two accession of G. herbaceum L. in response to drought
Source: BMC Genomics. 2012 Mar 16;13:94. doi: 10.1186/1471-2164-13-94 (PMC3320563; doi:10.1186/1471-2164-13-94)
Supplement: Additional file 16 — Correlation analysis between differentially expressed genes obtained in microarray and contigs obtained from transcriptome sequencing. PPT file containing Pearson correlation graph between microarray and contigs of transcriptome sequencing. For each contigs the counts were converted to transcripts per million which was then converted to log2 counts and their ratio was calculated for fold change between Vagad and RAHS-14. [file 1471-2164-13-94-S16.PPT]

## Slide 1
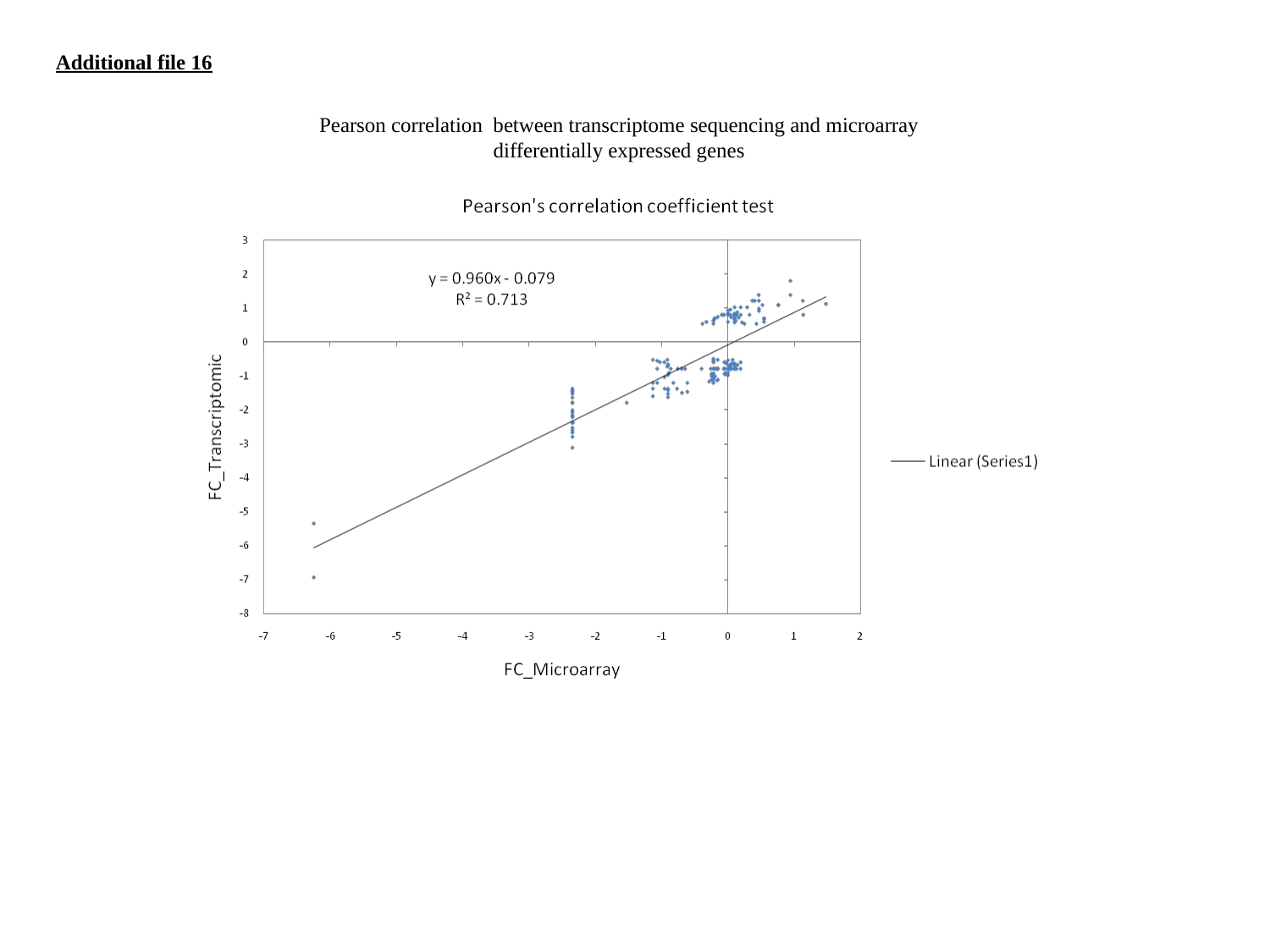

Additional file 16
Pearson correlation between transcriptome sequencing and microarray differentially expressed genes
